# The long noncoding RNA CTA‐941F9.9 is frequently downregulated and may serve as a biomarker for carcinogenesis in colorectal cancer

**DOI:** 10.1002/jcla.22986

**Published:** 2019-07-25

**Authors:** Zhexu Guo, Cen Zhou, Xi Zhong, Jinxin Shi, Zhonghua Wu, Kaiwen Tang, Zhenning Wang, Yongxi Song

**Affiliations:** ^1^ Department of Surgical Oncology and General Surgery The First Hospital of China Medical University Shenyang China

**Keywords:** biomarker, carcinogenesis, colorectal cancer, CTA‐941F9.9, long noncoding RNAs

## Abstract

**Background:**

Long noncoding RNAs (lncRNAs) participate in the carcinogenesis of many different cancers. This study aimed to detect expression of lncRNA CTA‐941F9.9 in colorectal cancer tissues compared with matched nontumorous adjacent tissues (NATs). Moreover, we investigated whether this molecule is able to influence carcinogenesis in colorectal cancer (CRC).

**Methods:**

Colorectal cancer tissues and NATs from two cohorts of patients were examined. Quantitative PCR was performed to quantify levels of CTA‐941F9.9 expression in these samples. The association between CTA‐941F9.9 expression and clinicopathological features, including receiver operating characteristic (ROC) curves, was also analyzed to evaluate the diagnostic value of CTA‐941F9.9 in CRC. Potential effects of lncRNA CTA‐941F9.9 on CRC cells were assessed via autophagy, transwell assay, CCK8 assays, and flow cytometry.

**Results:**

Our experimental results showed lncRNA CTA‐941F9.9 to be significantly downregulated in CRC tissues in both cohorts, with areas under the ROC curve (AUC) of 0.802 and 0.876. However, no significant correlations between CTA‐941F9.9 expression levels and clinicopathological characteristics or patient outcomes were observed. We also found that CTA‐941F9.9 promotes autophagy in CRC cell lines but no significant function of CTA‐941F9.9 in regulating cancer cell proliferation or migration.

**Conclusions:**

LncRNA CTA‐941F9.9 is frequently downregulated in CRC compared with NATs and might play an important role in CRC carcinogenesis.

## 1 INTRODUCTION

In 2018, almost 1.8 million cases and 881 000 deaths globally were recorded for colorectal cancer (CRC), the third most prevalently diagnosed cancer and second in terms of mortality, accounting for 10% of all cancer deaths.^1^ In China, CRC ranks among the top five most diagnosed cancers and types associated with mortality.^2^ Although medical technology and treatment strategies have strongly progressed, 60% of CRC patients are still diagnosed at advanced stages, and only 5% of patients with distant metastasis survive for more than 5 years.^3^ Therefore, it is critical to identify novel diagnostic biomarkers that can contribute to the diagnosis of CRC.

Long noncoding RNAs (lncRNAs) consist of more than 200 nucleotides but have no capacity to encode proteins.^4^, ^5^ Over the past few years, growing evidence has suggested that lncRNAs may be associated with the progression of numerous diseases.^6^ For example, lncRNA‐KRTAP5‐AS1 and lncRNA‐TUBB2A can promote cell proliferation, invasion, and EMT in gastric cancer cell lines; lnc‐XLEC1 is downregulated in endometrial carcinoma and associated with its incidence and prognosis.^7^ High expression of lnc‐SNHG15 in CRC patients correlates with lymph node and liver metastases, and patients with high lncRNA‐SNHG15 expression have a shorter median overall survival.^8^ Moreover, downregulation of lncRNA MEG3 indicates poor prognosis in CRC, and by regulating cell proliferation, MEG3 has been demonstrated to participate in the onset and progression of CRC.^9^ Qi et al^10^ also indicated that lncRNA might be used as a biomarker for the early detection of metastasis in CRC. As lncRNA dysregulation plays a crucial role in CRC development, lncRNAs are regarded as novel biomarkers and therapeutic targets for CRC patients.

LncRNA CTA‐941F9.9 (ENSG00000238120) is a novel lncRNA of 548 nucleotides expressed from chromosome 22. However, the level of CTA‐941F9.9 expression between CRC tissues and matched NATs has not been reported. Therefore, this study aimed to quantify CTA‐941F9.9 expression in CRC tissues and NATs and to evaluate the biological function of CTA‐941F9.9 in carcinogenesis processes in CRC.

## 2 MATERIAL AND METHODS

### 2.1 Ethical approval of the study protocol

This study was conducted according to the principles established in the Declaration of Helsinki. Informed consent forms were signed by all participants before experimental samples were extracted. The informed consent of the participants and the institutional ethical principles were reviewed and authorized by the Research Ethics Committee of China Medical University.

### 2.2 Tissue samples

All tissue samples were obtained from First Hospital of China Medical University. One cohort consisted of 74 patients who received radical resection surgery for CRC in 2010; the second comprised 59 patients who underwent surgery from December 2015 to March 2016. CRC was histopathologically confirmed in all patients. Pair‐matched tissues were obtained from an area more than 5 cm away from the lesions. All tissue samples were snap‐frozen in liquid nitrogen and stored at −80°C before use. No patient received preoperative chemotherapy or radiotherapy prior to resection. Tumor histological grades were assessed using World Health Organization guidelines as the standard criterion. The eighth edition of the International Union Against Cancer tumor‐node‐metastasis (TNM) staging system was applied for classifying pT (depth of invasion) and pN (primary node). Follow‐up investigations were performed every 3‐6 months after surgery. The overall survival (OS) time was considered as the interval between surgery and the last follow‐up investigation or death.

### 2.3 RNA isolation and reverse transcription

Total RNA was extracted from CRC tissues and NATs using TRIzol (Invitrogen) according to the manufacturer's instructions. Total RNA was solubilized in RNase‐free dH2O, and RNA concentration and purity were measured using a nanophotometer UV/Vis spectrophotometer (A260/A280 between 1.8 and 2.0) (Implen, GmbH). Reverse transcription was performed using PrimeScript™ RT Reagent Kit with gDNA Eraser according to the manufacturer's instructions (TaKaRa). In brief, a 10 μL reaction mixture containing 1 μL of gDNA Eraser, 2 μL of 5× gDNA Eraser Buffer, and 1 μg of total RNA (diluted to 7 μL using RNase‐free dH2O) was incubated at 42°C for 2 minutes in a GeneAmp PCR 9700 Thermocycler (Applied Biosystems Life Technologies). A 10 μL reaction mixture containing 1 μL of PrimeScript RT Enzyme Mix1, 4 μL of 5× PrimeScript Buffer 2 (for real‐time PCR), 1 μL of RT Primer Mix, and 4 μL of RNase‐free water was added, and the samples were then incubated in a PCR thermocycler for 15 minutes at 37°C and 5 seconds at 85°C.

### 2.4 Real‐time PCR

SYBR^®^ Premix Ex Taq II (Takara) was used for quantitative real‐time PCR with a Light Cycler 480 II Real‐Time PCR system (Roche Diagnostics). Each 25 μL reaction mixture for amplifying CTA‐941F9.9 contained 12.5 μL of SYBR, 0.5 μL of forward primer, 0.5 μL of reverse primer, 9.5 μL of RNase‐free water, and 2 μL of cDNA, which was synthesized by reverse transcription. The reaction was performed in a LightCycler^®^480 Multiwell Plates 96 (Roche Diagnostics) using 1 cycle at 95°C for 30 seconds, followed by 45 cycles at 95°C for 5 seconds, and 60°C for 30 seconds. The primers used for quantitative real‐time PCR were as follows: CTA‐941F9.9 primer F, 5′–CTACGGTGGCTCCGTTTCTT–3′, and R, 5′–ACGTTTCCCCACATCGTTCA–3′; GAPDH primer F, 5′–CGGATTTGGTCGTATTGGG–3′, and R, 5′–CTGGAAGATGGTGATGGGATT–3′ (Sangon Biotech). All real‐time PCRs were performed in triplicate.

### 2.5 Cell culture, plasmid construction, and cell transfection

Five CRC cell lines and one normal colonic epithelial cell line were purchased from American Type Culture Collection. Three lines (HCT116, HT29, and RKO) were cultured in an environment containing 5% CO_2_ at 37°C with RPMI 1640 medium supplemented with 10% fetal bovine serum. SW620 and SW480 cell lines were cultured under the same conditions without CO_2_. FHC cells were grown in DMEM:F12 medium instead of RPMI 1640. Full‐length CTA‐941F9.9 was cloned into the pcDNA3.1 expression vector (GenePharma) for overexpression in the cell lines. Three different siRNAs (Ribobio) were designed for CTA‐941F9.9 knockdown. All transfection procedures followed protocols provided by the manufacturer.

### 2.6 Western blotting analysis

All transfected colorectal cancer cells were collected and lysed using Total Protein Extraction Kit (KeyGen Biotech). Total proteins (30 μg/lane) were separated by 12% SDS‐polyacrylamide gel (SDS‐PAGE) and then transferred onto PVDF membranes (Millipore). The membranes were incubated overnight at 4°C with primary antibodies, including anti‐LC3B (Abcam, ab51520) and anti‐beta actin (Abcam, ab6276). After immunoblotting with peroxidase‐conjugated Affinipure goat anti‐mouse IgG or peroxidase‐conjugated Affinipure goat anti‐rabbit IgG, proteins were detected by GelCapture version software (DNR Bio‐Imaging Systems).

### 2.7 Cell proliferation assay

Cell Counting Kit‐8 (CCK‐8, Dojindo) was used to measure cell proliferation potential. HCT116 cells (3 × 10^3^) or RKO cells were seeded in 96‐well plates and cultured with medium containing serum. At 24, 48, 72, and 96 hours, 10 μL reagent was added and incubated for 1 hour at 37°C, and absorbance at 450 nm was measured using a microplate reader (Spectra Max plus384; Molecular Devices).

### 2.8 Transwell assay

To verify the migration capacity of CRC cells, a sample of 1.0 × 10^5^ CRC cells was seeded into the upper chamber of transwell devices with 200 μL RPMI‐1640 medium. A total of 700 μL of RPMI‐1640 medium including 10% FBS was added to the lower chamber. After incubating at 37°C for 48 hours, the cells in the upper chamber were gently removed. After fixing with pure methanol for 1 minute, the insert was stained with hematoxylin for 3 minutes and then eosin for 30 seconds. The average number of migratory cells was counted in ten randomly chosen visual fields under an inverted microscope (Leica DMI300B).

### 2.9 Flow cytometry

Single‐cell suspensions were generated following trypsinization and resuspension. Before cell cycle experiments, CRC cells were fixed in 70% ice‐cold ethanol for at least 2 hours and then stained with propidium iodide according to the manufacturer's protocol. For apoptosis analysis, cells were stained with Annexin V‐APC and propidium iodide using Annexin V‐APC Apoptosis Detection Kit (KeyGEN). All samples and statistics were analyzed using a FACS caliber flow cytometer (BD Biosciences), BD Cell Quest software, and LSRFortessa (BD Biosciences).

### 2.10 Statistical analysis

All statistical analyses were performed using SPSS software version 21.0 (IBM) and GraphPad Prism 5 (GraphPad Software). We used the 2^−ΔΔ^ *^C^* ^t^ method to calculate expression of lncRNA in cancer tissues compared with NATs (ΔΔCt = ΔCt_tumor CTA‐941F9.9_ − ΔCt_NAT CTA‐941F9.9_). ΔCt indicates the difference of the *C*_t_ value between the target and endogenous reference (GAPDH; Δ*C*_t_ = *C*_tCTA‐941F9.9_ − *C*_tGAPDH_).^11^ Statistical differences in expression of lncRNA CTA‐941F9.9 in CRC tissues compared with NAT controls were measured by Student's *t* test. The Mann‐Whitney *U* test (for two groups) and Kruskal‐Wallis test (for at least three groups) were used to detect associations between CTA‐941F9.9 expression levels and clinicopathological features. The Spearman correlation coefficient was employed to evaluate the association between CTA‐941F9.9 expression levels and clinicopathological characteristics (age and tumor size). The Kaplan‐Meier method was utilized to evaluate survival curves and the log‐rank test to determine statistical discrepancy between survival times. Two ROC curves were generated to measure the diagnostic value of lncRNA levels. Differences with a two‐tailed *P*‐value <.05 were considered statistically significant.

## 3 RESULTS

### 3.1 CTA‐941F9.9 is downregulated in CRC tissues

**Figure 1:**
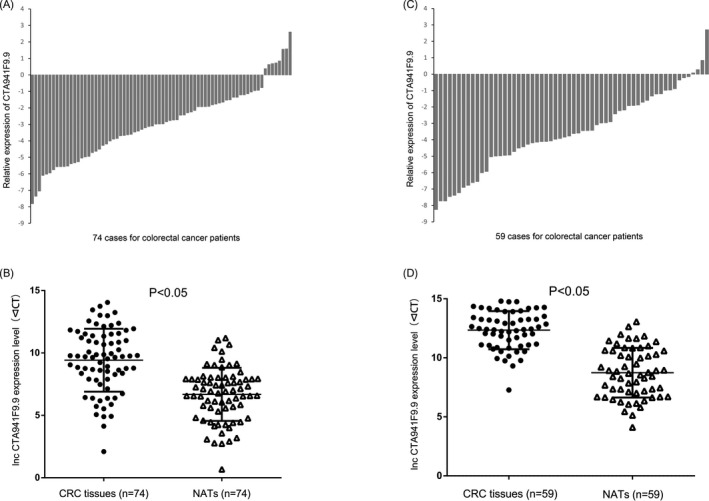
The expression levels of lncRNA CTA‐941F9.9 in colorectal cancer (CRC) A and B. Relative expression of long noncoding RNA (lncRNA) CTA‐941F9.9 in 74 human CRC tumorous tissues and their paired nontumorous adjacent tissues. C and D, Relative expression of lncRNA CTA‐941F9.9 in 59 human CRC tumorous tissues and their paired nontumorous adjacent tissues

The expression levels of CTA‐941F9.9 were measured by real‐time PCR between CRC tissue samples and NATs, and the results showed that CTA‐941F9.9 was downregulated in 89.2% (66/74) of CRC tissues compared to their NATs (*P* < .001) (Figure 1A and B). To confirm the expression trend of CTA‐941F9.9, another cohort of 59 CRC tissue samples and paired NATs from patients who underwent surgery in the past 3 years were measured. This cohort also revealed CTA‐941F9.9 downregulation in 93.2% (55/59) of CRC tissues compared with NATs (Figure 1C and D). CTA‐941F9.9 expression levels were similar in both cohorts; thus, CTA‐941F9.9 expression was markedly decreased in CRC tissues compared with NATs. We also tested correlations between CTA‐941F9.9 expression and clinicopathological characteristics and found no significant correlation between CTA‐941F9.9 expression levels and age, sex, tumor location, tumor size, histological grade, pT stage, pN stage, or TNM stage for the first (Table 1) or second (Table 2) cohort.

**Table 1:** Relationship of clinicopathological features with CTA‐941F9.9 expression in cohort 1 tissue samples of colorectal cancer

Characteristics	Number of patients (%)	CTA‐941F9.9 expression level^a^	*P*‐Value
Age			
Maximum‐Minimum (28‐83)	74 (100.0)	0.127 (0.040‐0.347)	.158
Gender			
Male	33 (44.6)	0.127 (0.066‐0.315)	.476
Female	41 (55.4)	0.128 (0.031‐0.371)	
Tumor location			
Colon	26 (35.1)	0.167 (0.037‐0.512)	.483
Rectum	48 (64.9)	0.156 (0.043‐0.304)	
Tumor size			
Maximum‐Minimum (2.3‐13.0 cm)	74 (100.0)	0.127 (0.040‐0.347)	.285
Histological grade			
Well	11 (14.9)	0.127 (0.090‐0.345)	.933
Moderately	59 (79.7)	0.128 (0.038‐0.391)	
Poorly	4 (5.4)	0.092 (0.027‐0.478)	
pT stage			
T2	10 (13.5)	0.156 (0.065‐0.347)	.799
T3	10 (13.5)	0.079 (0.043‐0.194)	
T4	54 (73)	0.149 (0.032‐0.401)	
pN stage			
0	44 (59.5)	0.151 (0.031‐0.391)	.868
1	24 (32.4)	0.101 (0.052‐0.304)	
2	6 (8.1)	0.159 (0.065‐0.554)	
pTNM stage			
1	7 (9.5)	0.184 (0.025‐0.345)	.870
2	37 (50.0)	0.150 (0.031‐0.422)	
3	30 (40.5)	0.111 (0.054‐0.297)	

a Median relative expression (25th‐75th percentile).

**Table 2:** Relationship of clinicopathological features with CTA‐941F9.9 expression in cohort 2 tissue samples of colorectal cancer

Characteristics	Number of patients (%)	CTA‐941F9.9 expression level^a^	*P*‐Value
Age			
Maximum‐Minimum (29‐86)	59 (100.0)	0.073 (0.032‐0.279)	.704
Gender			
Male	31 (52.5)	0.056 (0.031‐0.279)	.242
Female	28 (47.5)	0.092 (0.039‐0.394)	
Tumor location			
Colon	26 (44.1)	0.083 (0.031‐0.280)	1.000
Rectum	33 (55.9)	0.066 (0.031‐0.304)	
Tumor size			
Maximum‐Minimum (2.3‐12.0 cm)	59 (100.0)	0.073 (0.032‐0.279)	.443
Histological grade			
Well	11 (18.6)	0.081 (0.017‐0.178)	.149
Moderately	42 (71.2)	0.065 (0.031‐0.280)	
Poorly	6 (10.2)	0.384 (0.063‐1.097)	
pT stage			
T2	5 (8.5)	0.017 (0.011‐0.330)	.351
T3	19 (33.2)	0.187 (0.032‐0.330)	
T4	35 (59.3)	0.060 (0.033‐0.264)	
pN stage			
0	32 (54.2)	0.068 (0.020‐0.253)	.420
1	17 (28.8)	0.134 (0.057‐0.435)	
2	10 (16.9)	0.055 (0.025‐0.643)	
pTNM stage			
1	5 (8.4)	0.017 (0.011‐0.330)	.581
2	27 (45.8)	0.073 (0.032‐0.266)	
3	23 (39.0)	0.118 (0.047‐0.435)	
4	4 (6.8)	0049 (0.032‐0.808)	

a Median relative expression (25th‐75th percentile).

### 3.2 Association between CTA‐941F9.9 expression levels and patient survival

**Figure 2:**
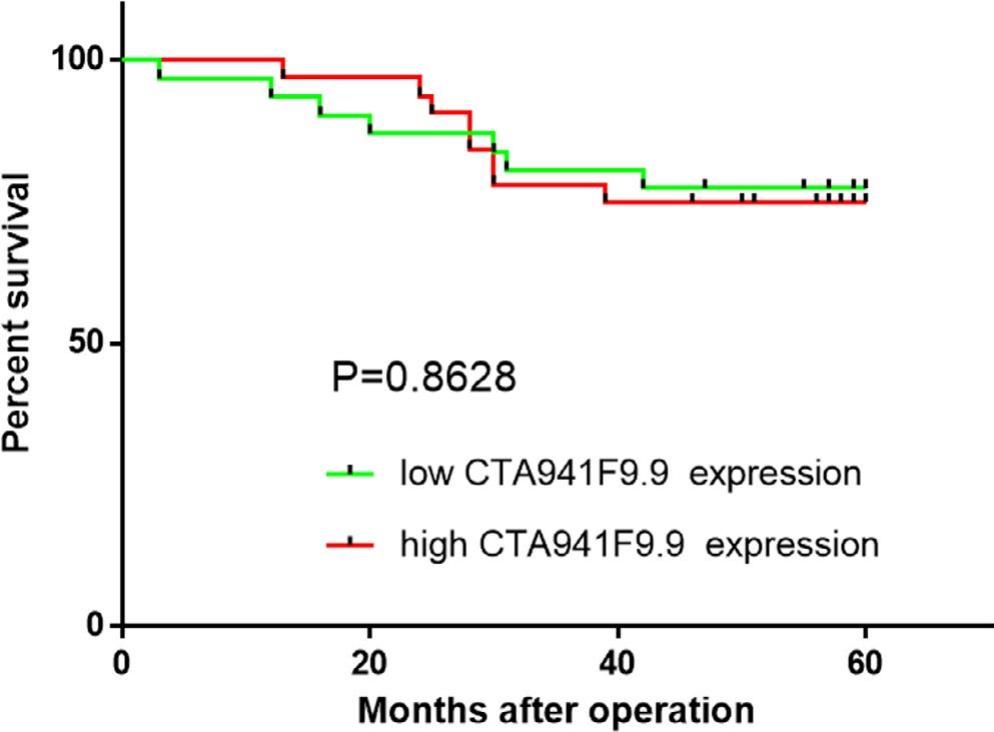
Kaplan‐Meier analysis of the correlation between lncRNA CTA‐941F9.9 expression level and overall survival

Kaplan‐Meier analysis was applied to examine whether CTA‐941F9.9 dysregulation is associated with patient survival time. After excluding 6 patients who were lost during follow‐up, the CRC patients were separated into two sets—high expression (n = 34) and low expression (n = 34)—according to median CTA‐941F9.9 expression levels. However, no significant difference in OS (*P* = .863) was observed between these two groups (Figure 2).

### 3.3 CTA‐941F9.9 is a potential diagnostic biomarker for CRC

**Figure 3:**
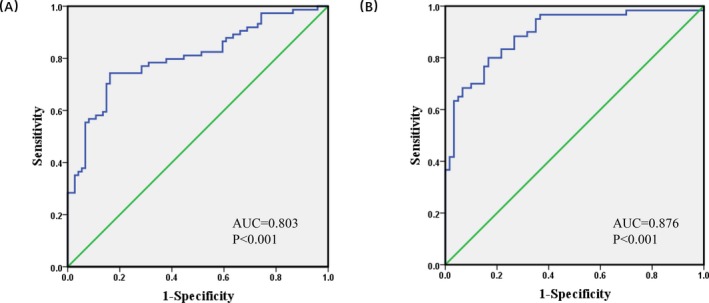
The receiver operating characteristic (ROC) curve of colorectal cancer (CRC) patients based on expression of long noncoding RNA (lncRNA) CTA‐941F9.9. A, The ROC curve of 74 CRC patients. B, The ROC curve of 59 CRC patients

We next constructed ROC curves to explore whether CTA‐941F9.9 can be used as a biomarker to distinguish CRC from normal tissues. Our results indicated an AUC of 0.803 (sensitivity, 74.3%; specificity, 83.8%) for cohort 1 and 0.876 (sensitivity, 78.2%; specificity, 85.1%) for cohort 2, suggesting that CTA‐941F9.9 has potential diagnostic value in CRC (Figure 3).

### 3.4 The effect of CTA‐941F9.9 on cell migration, cell proliferation, the cell cycle, and apoptosis

**Figure 4:**
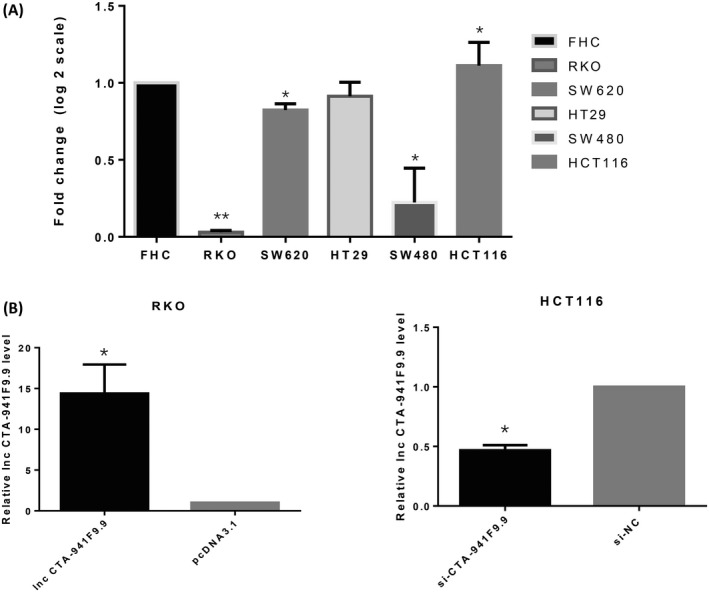
A, Expression of lncRNA CTA‐941F9.9 in CRC cell lines. B, Overexpression and knockdown efficiencies of lncRNA CTA‐941F9.9 in HCT 116 cells and RKO cells

**Figure 5:**
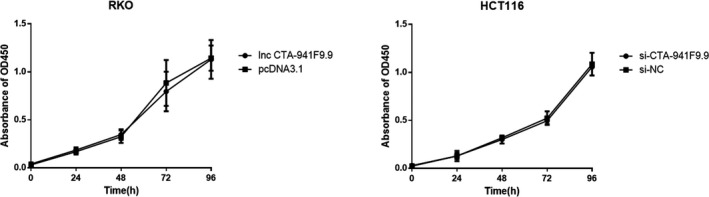
Daily assessment of overexpressing (lncRNA CTA‐941F9.9 group), silencing (si‐CTA‐941F9.9 group), and control group (pcDNA3.1/si‐NC) CRC cell proliferation for 4 d using a cell counting kit 8 (CCK8) assay

**Figure 6:**
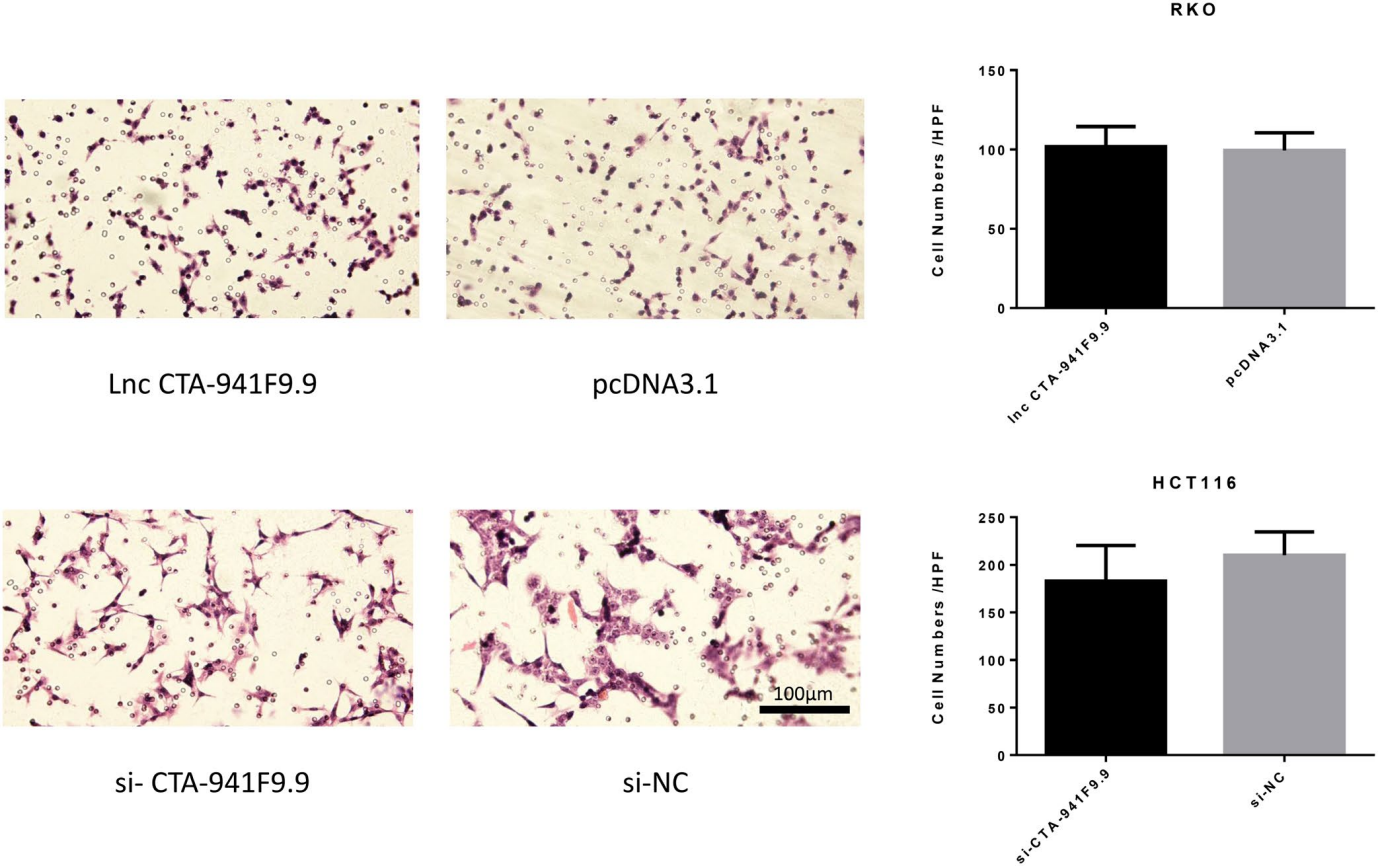
Exploration of lncRNA CTA‐941F9.9’s involvement in CRC cell migration

**Figure 7:**
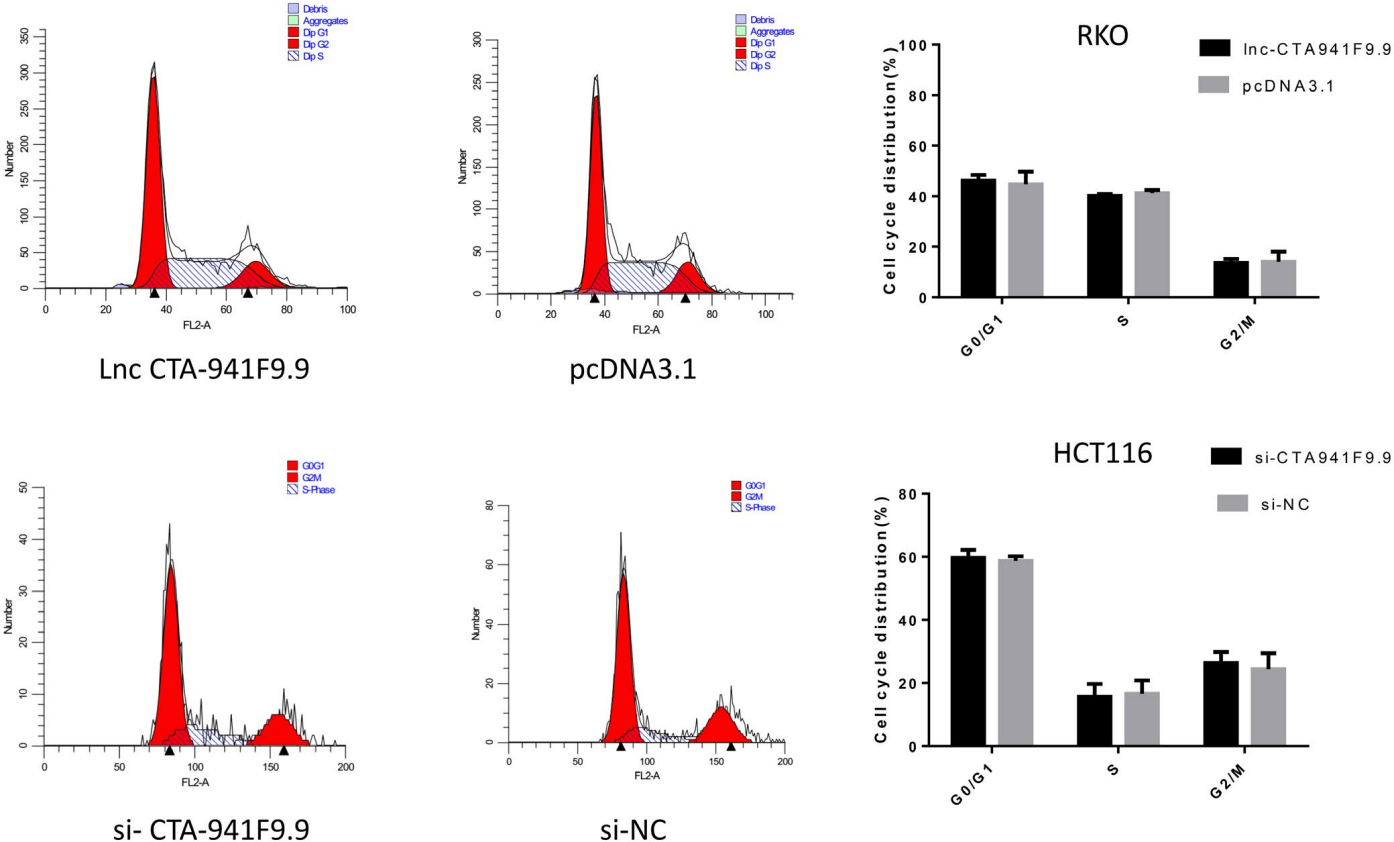
FACS analysis to detect the cell cycle after overexpressing or knocking down lncRNA CTA‐941F9.9

**Figure 8:**
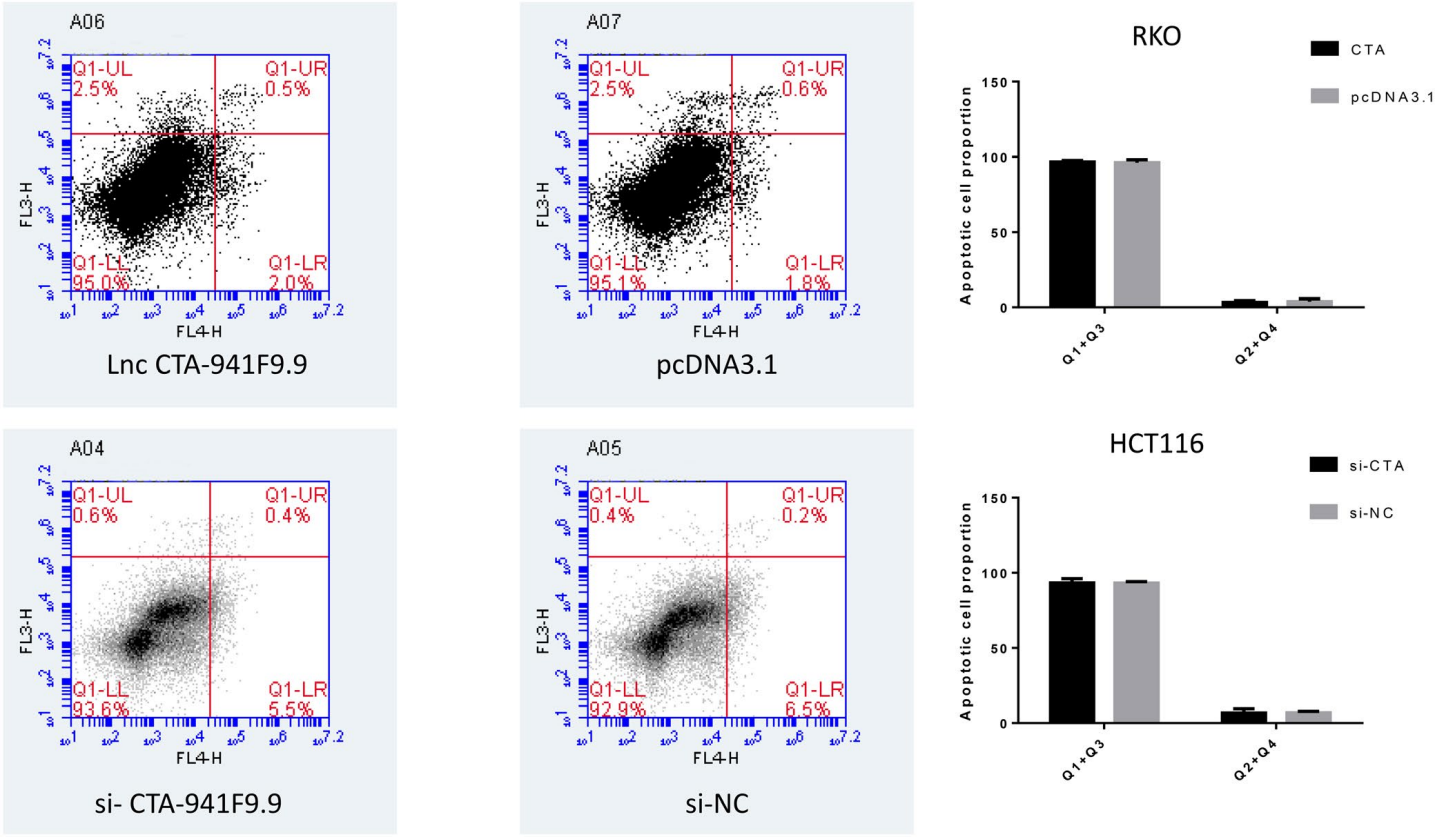
FACS analysis to detect the proportion of apoptotic cells after overexpressing or knocking down lncRNA CTA‐941F9.9

We next investigated the function of CTA‐941F9.9 in CRC progression by examining relative expression levels of CTA‐941F9.9 in six CRC cell lines. RKO cells exhibited the lowest expression of CTA‐941F9.9 and HCT116 cells the highest relative expression, with the other cell lines showing moderate expression (Figure 4A). Thus, we selected HCT116 cells for CTA‐941F9.9 silencing, termed the si‐CTA‐941F9.9 group, and selected RKO cells for CTA‐941F9.9 overexpression, termed the CTA‐941F9.9 group. The transfection efficiency was measured and recorded at 48 hours after transfection (Figure 4B). To verify the effect of CTA‐941F9.9 on cell proliferation, we performed a CCK‐8 assay and found no significant differences between the CTA‐941F9.9 and negative control (NC) groups or between the si‐CTA‐941F9.9 and si‐NC groups. We next conducted a transwell experiment to assess the effect of CTA‐941F9.9 on CRC cell migration and observed no significant difference after overexpressing or silencing CTA‐941F9.9. As also indicated by the results of cell cycle and cell apoptosis detection, no significant differences were obtained with overexpression or silencing of CTA‐941F9.9 (Figures 5, 6, 7, 8).

### 3.5 CTA‐941F9.9 promotes autophagy in CRC cell lines

**Figure 9:**
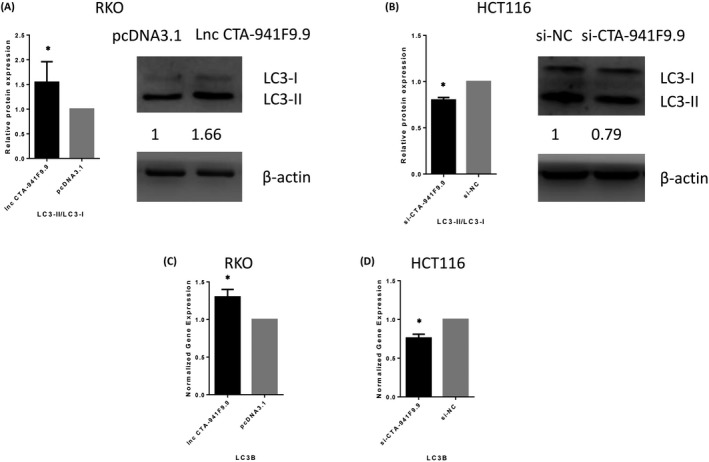
A and B, Protein expression profile of LC3‐II/LC3‐I after overexpressing or knocking down lncRNA CTA‐941F9.9. C and D, Gene expression profile of LC3B after overexpressing or knocking down lncRNA CTA‐941F9.9. qPCR results were normalized to GAPDH. Data are shown as the mean ± SD, n = 3. *Represents *P* < .05

Real‐time PCR and western blot analyses were performed to detect the impact of CTA 941F9.9 on CRC cell autophagy, showing that the ratio of LC3‐II/LC3‐I was markedly increased in CTA 941F9.9‐overexpressing cells and decreased in si‐CTA 941F9.9 cells. These results indicate that at the protein level, CTA 941F9.9 promotes expression of LC3‐II, a key protein of autophagy, and therefore promotes autophagy. In addition, real‐time PCR experiments indicated that CTA 941F9.9 promotes LC3B gene transcription and autophagy at the mRNA level (Figure 9).

## 4 DISCUSSION

Colorectal cancer is a highly heterogeneous disease involving numerous genetic and epigenetic alterations.^12^ Although medical technologies and treatment strategies have rapidly progressed, more than 60% of colorectal patients are still diagnosed at advanced stages and have poor outcomes.^3^ Therefore, detecting CRC at early stages is essential. In recent decades, CRC has generally been diagnosed using fecal occult blood testing (FOBT) and colonoscopy.^13^, ^14^ In the original trials of the FOBT screening test conducted in the 1980s, adherence to FOBT was only 67%, with low detection accuracy for CRC.^13^, ^15^ Similarly, colonoscopy has limitations including high cost and ineffectiveness in large population screening programs. CEA, CA19‐9, and CA72‐4 are the most commonly used serological biomarkers for diagnosing CRC; however, they lack sufficient accuracy. Thus, the identification of biomarkers with high specificity and sensitivity for early CRC screening might significantly improve CRC detection and patient outcomes.

NcRNAs have historically been considered nonfunctional, originally labeled as ‘junk RNAs’. However, research has revealed roles for lncRNAs and indicated that several are aberrantly expressed in many types of cancers.^16^ For example, CCAT1, a well‐known lncRNA, is upregulated in CRC, gastric cancer (GC), hepatocellular carcinoma, breast cancer, and ovarian cancer.^17^, ^18^, ^19^, ^20^, ^21^ Moreover, Sana et al^22^ reported that lncRNA‐uc.73 and lncRNA‐uc.388 are significantly decreased in CRC tissues and that both lncRNAs are potential diagnostic and prognostic biomarkers in CRC. Wang et al^23^ used microarray analysis and RT‐qPCR to show that CTA‐941F9.9 was strongly downregulated in gallbladder carcinoma, consistent with our study. Additionally, Liu et al^24^ found that CRNDE‐h is a potential diagnostic biomarker in CRC (AUC = 0.757). Our results indicate that lncRNA CTA‐941F9.9 is significantly downregulated in CRC tissues compared to NATs. Moreover, ROC curves yielded an AUC of 0.802 and 0.876, indicating that CTA‐941F9.9 is a good potential biomarker for CRC.

It is noteworthy that dysregulated lncRNAs in plasma, urine, gastric juice, and cerebrospinal fluid have the potential to reveal tumors at an early stage. Dong et al^25^ found that a combination of three circulating lncRNAs (CUDR, LSINCT‐5, and PTENP1) in serum had a significantly higher diagnostic value than did any single‐factor index, including CA19‐9 or CEA, reporting that the combination of three circulating lncRNAs yielded a new complementary marker for GC. Moreover, according to Wang et al,^26^ MALAT‐1 in urine is a promising biomarker for predicting prostate cancer, and Zheng et al^27^ demonstrated that UCA1 levels are increased in the gastric juice of GC patients compared with normal individuals. These studies demonstrate that lncRNAs are common in bodily fluids and therefore may be used as diagnostic biomarkers. Because of the low risk and simple collection compared with colonoscopy, these biomarkers may be of practical use in large population screening programs. Overall, the identification of suitable diagnostic biomarkers in the plasma of CRC patients might improve CRC detection and outcomes of patients. Future studies should focus on detecting the expression levels of CTA‐941F9.9 in plasma.

Autophagy works as a evolutionarily conservative degradation pathway in which damaged proteins and cytoplasmic components are removed, digested, and recycled to maintain cellular homeostasis and sustain viability under adverse conditions.^28^ The processes of autophagy can play both positive and negative role in cancer cells and may be crucial in pathophysiology of CRC.^29^ LncRNAs, as important regulatory factors, may positively regulate the autophagy process and negatively modulate autophagy. As important autophagy‐related lncRNAs are being discovered, such as HOTAIR, HULC, MALAT1, and GAS5,^30^, ^31^, ^32^, ^33^ lncRNAs are becoming increasingly important in the field of autophagy research. In general, lncRNAs will be the focus of future autophagy‐related research, and the regulatory role and function of lncRNA in autophagy will become increasingly prominent. The increase in CTA941F9.9 expression observed in CRC cells might enhance the levels of LC3, as demonstrated by our western blotting results, which indicates that CTA941F9.9 might promote autophagy in CRC. Wei et al^34^ found that FAT4 was downregulated in CRC tissues and promoted autophagy in CRC cells. Processed LC3 participates in the formation of autophagosomes and used to monitor autophagic activity. This protein includes two forms: LC3‐I and LC3‐II. The former is cytosolic and conjugated to phosphatidylethanolamine to form the latter which present both inside and outside autophagosomal membranes.^35^, ^36^ Besides, LC3‐II might regulate the formation of autophagosomes and manage their amount during autophagy.^37^, ^38^

Increasing evidence indicates that lncRNAs can regulate cancer cell proliferation, invasion, and metastasis.^39^, ^40^, ^41^ Our study aimed to investigate the function of CTA‐941F9.9 in CRC cancer cells, and we found that it has no specific function in regulating the cancer cell cycle, proliferation, migration, or apoptosis. Wang et al^42^ reported that CTA‐941F9.9 is likely to play important roles in the chondrogenic differentiation process, providing new insight into its regulatory function.^43^ The potential capacity of CTA‐941F9.9 to promote CRC cell differentiation remains to be investigated. CTA‐941F9.9 may also participate in the process of carcinogenesis.

The molecular mechanisms underlying CRC development are still unknown. As cancer is a complex disease with multiple factors and phases, the decrease in CTA‐941F9.9 expression in CRC may be affected by complex cancer‐related interactions. Future studies will help elucidate these interactions in CRC.

## 5 CONCLUSION

In conclusion, lncRNA CTA‐941F9.9 is frequently downregulated in CRC compared with NATs and might play an important role in CRC carcinogenesis.

## CONFLICT OF INTEREST

All authors have completed the ICMJE uniform disclosure form at www.icmje.org/coi_disclosure.pdf and declare no support from any organization for the submitted work; no financial relationships with any organizations that might have an interest in the submitted work in the previous three years; and no other relationships or activities that could appear to have influenced the submitted work.
